# Stacked mutations in wheat homologues of rice *SEMI-DWARF1* confer a novel semi-dwarf phenotype

**DOI:** 10.1186/s12870-024-05098-1

**Published:** 2024-05-09

**Authors:** Barbora Ndreca, Alison Huttly, Sajida Bibi, Carlos Bayon, George Lund, Joshua Ham, Rocío Alarcón-Reverte, John Addy, Danuše Tarkowská, Stephen Pearce, Peter Hedden, Stephen G. Thomas, Andrew L. Phillips

**Affiliations:** 1 Rothamsted Research, Harpenden, Hertfordshire AL5 2JQ UK; 2 Nuclear Institute for Agriculture and Biology, Faisalabad, Punjab Pakistan; 3 Laboratory of Growth Regulators, Institute of Experimental Botany, Czech Academy of Sciences and Palacky University, Šlechtitelů 27, Olomouc, CZ 78371 Czech Republic

**Keywords:** Wheat, Gibberellin, Dwarfing alleles, TILLING, Green revolution

## Abstract

**Background:**

Semi-dwarfing alleles are used widely in cereals to confer improved lodging resistance and assimilate partitioning. The most widely deployed semi-dwarfing alleles in rice and barley encode the gibberellin (GA)-biosynthetic enzyme GA 20-OXIDASE2 (GA20OX2). The hexaploid wheat genome carries three homoeologous copies of *GA20OX2*, and because of functional redundancy, loss-of-function alleles of a single homoeologue would not be selected in wheat breeding programmes.

Instead, approximately 70% of wheat cultivars carry gain-of-function mutations in *REDUCED HEIGHT 1* (*RHT1*) genes that encode negative growth regulators and are degraded in response to GA. Semi-dwarf *Rht-B1b* or *Rht-D1b* alleles encode proteins that are insensitive to GA-mediated degradation. However, because *RHT1* is expressed ubiquitously these alleles have pleiotropic effects that confer undesirable traits in some environments.

**Results:**

We have applied reverse genetics to combine loss-of-function alleles in all three homoeologues of wheat *GA20OX2* and its paralogue *GA20OX1* and evaluated their performance in three years of field trials. *ga20ox1* mutants exhibited a mild height reduction (approximately 3%) suggesting *GA20OX1* plays a minor role in stem elongation in wheat. *ga20ox2* mutants have reduced GA_1_ content and are 12–32% shorter than their wild-type segregants, comparable to the effect of the *Rht-D1b* ‘Green Revolution’ allele. The *ga20ox2* mutants showed no significant negative effects on yield components in the spring wheat variety ‘Cadenza’.

**Conclusions:**

Our study demonstrates that chemical mutagenesis can expand genetic variation in polyploid crops to uncover novel alleles despite the difficulty in identifying appropriate mutations for some target genes and the negative effects of background mutations. Field experiments demonstrate that mutations in *GA20OX2* reduce height in wheat, but it will be necessary to evaluate the effect of these alleles in different genetic backgrounds and environments to determine their value in wheat breeding as alternative semi-dwarfing alleles.

**Supplementary Information:**

The online version contains supplementary material available at 10.1186/s12870-024-05098-1.

## Background

Plant height is an important trait in small-grain cereal crops: although stature, as a component of total biomass, contributes to grain yield, taller plants are susceptible to lodging under high winds or heavy rain, resulting in loss of harvestable yield and quality [1]. The application of high levels of nitrogen fertilisers promotes growth and thereby increases the frequency and severity of lodging. The success of the ‘Green Revolution’ in the second half of the twentieth century thus depended on the introduction of semi-dwarfing alleles into the major cereals to counteract the growth-promoting effects of the increased application of fertiliser.

In common wheat (*Triticum aestivum* L.) two semi-dwarfing alleles, *Rht-B1b* and *Rht-D1b* were sourced from the experimental Japanese variety ‘Norin 10’ (reviewed in [2]). Although *Rht-B1b* and *Rht-D1b* reduce growth and, hence, biomass, the reduced investment in culm growth results in increased grain numbers and a higher harvest index, despite a decrease in grain size, resulting in similar or even increased yield compared with tall lines [3, 4]. Since the introduction of these alleles into breeding programmes in the USA and, most notably, at CIMMYT in Mexico, *Rht-B1b* or *Rht-D1b* are now found in > 70% of commercial wheat varieties in some regions [5]. These alleles are usually used singly as the presence of semi-dwarfing alleles in both genes together results in excessive reduction in growth, with consequential loss of biomass and yield [6].

Wild-type *Rht1* encodes a DELLA protein, a growth-suppressing nuclear component of the signal transduction pathway of the phytohormone gibberellin (GA) [7]. DELLA proteins suppress growth by modulating the activity of target transcription factors through protein–protein interactions, such as those of the INDETERMINATE DOMAIN family [8]. The interaction between bioactive forms of GA and the receptor protein GIBBERELLIN INSENSITIVE DWARF1 (GID1) triggers a conformational change that enhances GID1’s affinity for DELLA proteins [9–11]. This interaction leads to the F-box protein-mediated polyubiquitination of DELLA proteins, tagging them for degradation by the E3-ubiquitin ligase complex, thereby releasing the suppression of growth. The dominant, gain-of-function semi-dwarfing alleles, *Rht-B1b* and *Rht-D1b*, encode proteins that are prematurely truncated in the DELLA domain that is required for interactions with GID1; translational re-initiation results in the production of N-terminally-truncated RHT1 proteins (ΔN-RHT1) that retain growth-suppression activity but no longer interact with GID1 and are thus insensitive to GA [12]. There is evidence that low efficiency of translational re-initiation results in the accumulation of only small amounts of ΔN-RHT1, yielding a semi-dwarf phenotype; higher production of GA-insensitive RHT1 proteins such as in *Rht-B1c*, which encodes a protein with a 30 amino acid insertion in the DELLA domain, confers a more severe dwarf phenotype [12, 13].

In addition to its role in promoting stem growth, GA is involved in a wide range of developmental processes, including germination, root growth, flowering, pollen development and grain size [14]. Unlike *Arabidopsis*, which has five DELLA genes with specific, but overlapping, roles in plant development and different expression patterns [15], the hexaploid wheat genome contains a homoeologous triad of *Rht1* genes that are expressed similarly across multiple organs and tissues [13]. As a result, the *Rht-B1b* and *Rht-D1b* semi-dwarfing alleles also confer a range of negative pleiotropic effects including reduced coleoptile and seedling root growth, reduced biomass, poor anther extrusion (relevant in the production of hybrid wheat) and reduced grain size [4, 6, 16, 17]. These pleiotropic effects have consequences for yield; for example, shorter coleoptiles result in poor emergence in dry environments that require deep seed drilling [18]. Therefore, there is considerable interest in identifying alternative dwarfing alleles for wheat that confer resistance to lodging while maintaining grain yield and avoiding some of the negative pleiotropic effects of *Rht-B1b* and *Rht-D1b* alleles.

**Fig. 1:**
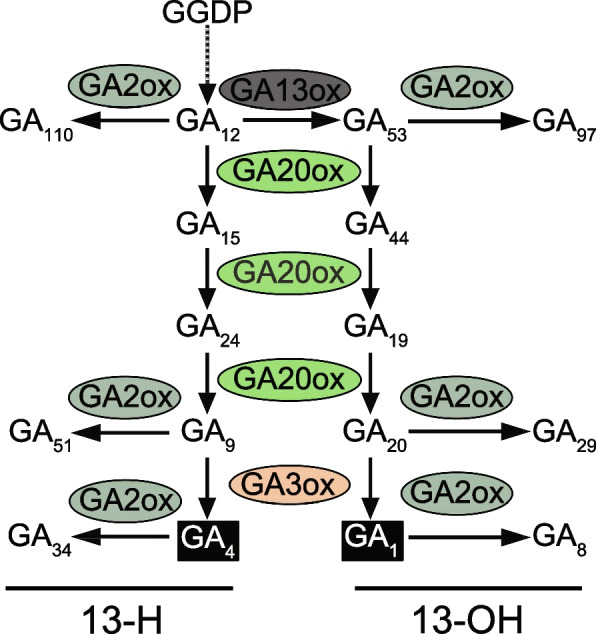
The final stages of the GA biosynthesis pathway in plants showing the reactions catalysed by 2-ODD enzymes. Depending on the activity of the cytochrome P450 mono-oxygenase GA 13-OXIDASE, the pathway proceeds either through the 13-hydroxylation (13-OH) or non-13-hydroxylation (13-H) branches. Bioactive GA_1_ and GA_4_ are highlighted in black boxes

In rice (*Oryza sativa*), which similarly saw large yield improvements during the ‘Green Revolution’, reduction in plant stature was achieved with alleles of *SEMIDWARF1* (*SD1*), first identified in the Taiwanese variety ‘Dee-geo-woo-gen’ (reviewed in [2]). These alleles are now widely deployed in rice breeding programmes. For example, a screen of 57 commercial rice varieties found that 38 carried *sd1* alleles [19]. *SD1* encodes GA 20-OXIDASE2 (GA20OX2), a 2-oxoglutarate-dependent dioxygenase (2-ODD) enzyme that catalyses three consecutive oxidation reactions at the C-20 position of precursor GAs, resulting in the loss of C-20 and leading to the formation of GA_9_ in the non-13-hydroxylation (13-H) pathway and GA_20_ in the 13-hydroxylation (13-OH) pathway (Fig. 1). In common with *GA 3-OXIDASE* (*GA3OX*) genes, expression of some *GA20OX* genes is promoted by DELLA proteins, and therefore reduced by GA action, as part of the mechanism for GA homeostasis [20]. Semi-dwarfing alleles of *SD1* contain mutations that impair or abolish enzyme activity, reducing bioactive GA levels in elongating stem tissues [21, 22]. GA 20-OXIDASE in rice and other cereals is encoded by a multigene family of four members with tissue-specific expression patterns [23] such that pleiotropic effects of *sd1* mutations are less widespread: for example, *sd1* semi-dwarfing alleles increase grain number and maintain yields without a negative effect on grain size [24].

The level of bioactive GA_1_ in the elongating stems of *sd1* mutants is only 20–35% lower than in wild-type plants, suggesting that other GA20OX enzymes contribute to GA biosynthesis in these tissues [22]. In rice, variation in the activity of *OsGA20OX1*, a paralog of *OsGA20OX2*, confers changes in height and panicle architecture, demonstrating that both these genes contribute to stem elongation in rice [25–27].

Therefore, loss-of-function alleles of *GA20OX2* and, potentially, *GA20OX1* are appealing targets for exploitation in wheat. However, while loss-of-function alleles of GA biosynthetic genes can be readily identified in diploid crop species through their effects on stature, in hexaploid wheat, the majority of homoeologous gene triads exhibit balanced expression profiles such that loss-of-function mutations in a single homoeologue may confer only a mild phenotypic effect [28, 29]. As a result of this functional redundancy, wheat germplasm carrying loss-of-function alleles in all three *GA20OX1* or *GA20OX2* homoeologues do not exist in nature and have never been subjected to selection during domestication or in formal breeding programmes.

In the current study, we exploited a chemically-mutagenized population of the UK spring wheat variety ‘Cadenza’ to develop lines carrying stacked loss-of-function alleles in all homoeologues of *GA20OX1* and *GA20OX2*. Across three years of field evaluations, we found that mutations in *GA20OX1* have only a mild effect on stem elongation. By contrast, stacked mutations in *GA20OX2* confer reductions in height comparable to those of the ‘Green Revolution’ *Rht-D1b* allele with no significant reduction in yield. Our results indicate the potential utility of *GA20OX2* genes as an alternative source of semi-dwarfing alleles for breeders, especially for those environments where *Rht1* alleles are unfavourable.

## Methods

### Plant materials and target genes

A population of approximately 6,000 M_1_ lines of the UK spring common wheat cultivar ‘Cadenza’ mutagenized with ethyl methanesulphonate (EMS) has been described previously [30]. For this work, an M_2_ population of 2,150 lines, each derived from an individual M_1_ plant, was grown; leaf material and M_3_ seeds were collected from the 2,057 fertile plants. Plant cultivation and propagation was as previously described [31] and DNA was extracted from 3–5 g leaf tissue samples [32]. Field experiments included wild-type ‘Cadenza’ and a line containing an introgressed *Rht-D1b* semi-dwarfing allele in the ‘Cadenza’ background described previously [12].

Wheat genes and their homoeologues encoding *GA20OX1* and *GA20OX2* have been previously identified [33]. A list of all genes from the GA biosynthetic, inactivation and signalling pathways, with updated gene IDs and gene names according to Boden et al. [34] is provided in Additional file 2, Table S1.

### Mutation discovery

Mutations in the target genes within the Cadenza-EMS population were identified from genomic DNA samples using either digestion of heteroduplex PCR products of target genes with the endonuclease *Cel*I [35], or by high-resolution melt analysis (HRM) [36]. Mutations in *TaGA20OX1-A1* and *TaGA20OX1-B1* were identified using the *Cel*I-based TILLING protocol essentially as described previously [35]. Briefly, DNA samples from individual Cadenza-EMS M_2_ lines were normalised to 100 ng/µL and pooled two-fold. After amplification of the complete gene sequence using homoeologue-specific primers (Additional file 2, Table S2), PCR products were incubated with a celery petiole extract containing *CelI* and separated on acrylamide gels on a LI-COR gen analyser. Candidates were confirmed by sequencing of PCR products from individual M_2_ lines using Hot-Shot Diamond PCR Mastermix supplied by Client Life Science (Stourbridge, UK).

Mutations in *TaGA20OX1-D1* and the three homoeologues of *TaGA20OX2* were identified by HRM as described previously [37]. Homoeologue-specific PCR products from 2x-pooled DNA samples were diluted 100-fold and re-amplified using HRM primers (Additional file 2, Table S2) in the presence of LCGreen dye (Client Life Science, Stourbridge, UK) to generate PCR products of 200–350 bp. Mutations were detected by melt analysis on an Idaho Technology LightScanner and confirmed by sequencing of PCR products from individual M_2_ lines. SIFT analysis was performed using the Variant Effect Predictor at https://plants.ensembl.org/Triticum_aestivum/Tools/VEP to predict the functional effect of induced amino acid changes on the encoded protein and whether an amino acid substitution is likely to affect protein function [38]. As these methods are laborious and low throughput, we screened just enough lines to identify at least one candidate loss-of-function mutant in each target gene.

### Heterologous expression of *GA20OX* mutants

For *GA20OX1-D1* and *GA20OX2-D1*, heterologous expression in *E. coli* was used to assess the effects of candidate mis-sense mutations on GA 20-oxidase activity. Synthetic cDNAs of wild-type and mutant variants of the two genes were synthesised (Genscript Biotech, Piscataway, NJ, USA) and inserted into pET32b (Merck, NJ, USA) for expression as C-terminal fusions to thioredoxin. Plasmids were transferred to *E. coli* BL21 Rosetta DE3 (Merck, NJ, USA); bacterial growth, induction, protein extraction and GA 20-oxidase assays with ^14^C-GA_12_ were carried out as described previously [33].

### Analysis of splicing in splice-site variants

The effects of splice-site mutations in *GA20OX1-A1b* and *GA20OX2-B1b* was investigated by deep sequencing of RT-PCR products. RNA was purified from flag leaf ligules and peduncular nodes of wild-type and mutant wheat lines at heading stage using a Monarch Total RNA Miniprep Kit (New England Biolabs, MA, USA). After pooling of replicate samples, 1 µg of each RNA was converted to cDNA using oligo dT_12-18_ as primer with Superscript III reverse transcriptase (Thermo Fisher Scientific, MA, USA). PCR was carried out using the RNA equivalent of ~ 200 ng cDNA using paralogue-specific primers (Additional file 2, Table S2) to amplify all three homoeologues in each case. Amplicons were sequenced on an Ion Torrent PGM sequencer. To allow barcoding of individual samples, primers used in the initial PCR were synthesised to include a 5’ M13 tail sequence on the forward primer and the Ion trP1 adaptor sequence on the reverse primer. In a second round of PCR, a forward primer consisting of the IonA adaptor sequence fused with IonXpress barcode sequence and the M13 sequence was used in conjunction with a reverse primer matching the IontrP1 adaptor to produce sequence-ready, barcoded amplicon libraries. After pooling and quantification on a Bioanalyzer 2100 (Agilent), libraries were templated using the Ion PGM HI-Q View OT2 kit (Thermofisher) and sequenced using the Ion PGM HI-Q View Sequencing kit (Thermofisher) with 400 bp reads. Sequence reads were mapped to the *GA20OX* reference genes using either bbmap [39] with parameters “maxindel = 2, subfilter = 2” or with Bowtie2 in Geneious (Biomatters Inc, Aukland, New Zealand) with both Gaps and Mismatches set at a maximum of 1%. The two approaches gave comparable results.

### Crossing and genotyping

To generate lines carrying mutations in each of the three homoeologues for each paralogue, M_4_ individuals carrying each mutation were crossed in two stages (i.e. A x B followed by AB x D), resulting in triple heterozygous (AaBbDd) BC_0_F_1_ lines. To remove excessive EMS mutations in the background, multiple triple BC_0_F_1_ individuals were crossed with wild-type ‘Cadenza’ to create 2–3 streams of BC_1_F_1_ lines (Additional file 1, Figure S1). After 3–4 further rounds of backcrossing, the resulting BC_4-5_F_1_ lines in each parallel stream were each selfed to generate the BC_4-5_F_2_ lines. Triple homozygotes, segregating wild-types and other allele combinations were selected from 376–564 individuals of the F_2_ populations in each backcrossing stream.

Mutations in crosses and in segregating populations were identified using KASP (Kompetitive Allele-Specific PCR) assays [40]. Genomic DNA was extracted and partially purified from 5 cm of seedling leaf using a bisulphite-based method [41] and dissolved in 200 µL TE0.1 (10 mM Tris–Cl pH8, 0.1 mM EDTA). KASP assays contained 2 µL (approximately 50 ng) gDNA, 4.2 pmol KASP common primer (Kc; Additional file 2, Table S2), 1.7 pmol wild-type allele-specific primer (Kw), 1.7 pmol mutant allele-specific primer (Km) and 5 µL KASP mastermix (LGC Biosearch Technologies, Teddington, UK) in a total volume of 10 µL. Reactions were run according to the manufacturer’s standard KASP protocol for 35–40 cycles preceded by 10 cycles of touchdown over either 61 °C-55°C or 68 °C-62°C. End-points of the assays were read on an Applied Biosystems 7500 Sequence Detection System and analysed using Klustercaller software (LGC Biosearch Technologies).

### Phenotypic characterisation

Mutant lines were initially characterised in a replicated trial in glasshouse conditions. The trial comprised backcrossed triple homozygous lines from each backcrossing stream (Additional file 1, Figure S1) containing *ga20ox1-A1b.B1b.D1b* (*ga20ox1*), *ga20ox2-A1b.B1b.D1b* (*ga20ox2(b)*), *ga20ox2-A1b.B1b.D1c* (*ga20ox2(c)*), *ga20ox2-A1b.B1b.D1d* (*ga20ox2(d*)) and their wild-type segregants at BC_4-5_F_2_ or BC_4-5_F_3_ stage. There were too few individuals to distinguish between the backcrossing streams for each triple mutant so analysis was carried out on data combined from the different streams. Up to 10 replicates of each line were germinated on damp filter paper, transferred to 2 cm × 2 cm cells of Rothamsted Prescription Mix compost with added nutrients [31] and subsequently moved to 15 cm pots in a randomised block design.

Field experiments grown at Rothamsted Experimental farm in Southeast England, were initially carried out in 1 m × 1 m replicated plots in a randomised block design sown in spring 2020 at a rate of 450 seeds/m^2^. Wild-type ‘Cadenza’ and lines containing an introgressed *Rht-D1b* semi-dwarfing allele in the ‘Cadenza’ background [12] were included for comparison. Two subsequent field trials were carried out on larger plots of 9 m × 2 m with four replicated randomised blocks, sown in spring 2021 and in autumn 2021, which was evaluated in 2022. All experiments were grown with standard farm practice for fertiliser and pesticides but with no plant growth regulators applied. Height measurements in the field in 2020 and 2021 were taken using a floating polystyrene disc of diameter 60 cm, taking six measurements per plot. Samples for yield, internode length and grain measurements were taken from the central 8 m × 1 m of each large plot to avoid edge effects. Internode lengths were taken on 10 individual tillers by measuring between the lower bounds of each node. Height values in 2022 were the sum of all internode measurements. Grain sizes were measured on samples of ~ 200 grain using a Videometerlab imager (Videometer, Herlev, Denmark). Grain yield was measured at final harvest at 85% dry matter, calculated using the fresh weight of grain per plot and the grain dry matter content. Dry matter was calculated from the fresh and dry weight of a sub-sample of approximately 80 g, dried for 16 h at 105 °C.

### Gibberellin quantification

Sample preparation and analysis of GAs was performed according to the method described in [42] with some modifications. Briefly, tissue samples of about 5 mg dry weight were ground to a fine powder using 2.7 mm zirconium oxide beads (Retsch GmbH & Co. KG, Haan, Germany) and a MM 400 vibration mill at a frequency of 27 Hz for 3 min (Retsch) with 1 mL of ice-cold 80% acetonitrile containing 5% formic acid as extraction solution. The samples were then extracted overnight at 4 °C using a benchtop laboratory rotator after adding 3 pmol of the following [17-^2^H_2_]-labelled GA standards: GA_1_, GA_4_, GA_9_, GA_19_, GA_20_, GA_24_, GA_29_, GA_34_ and GA_44_ purchased from OlChemIm, Czech Republic. The homogenates were centrifuged at 37,000 × *g* at 4 °C for 10 min, the supernatants further purified using reversed-phase and mixed mode SPE cartridges (Waters, Milford, MA, USA) and analysed by ultra-high performance liquid chromatography-tandem mass spectrometry (UHPLC-MS/MS) with a Xevo TQ-XS triple quadrupole mass spectrometer (Waters Milford, MA, USA). GAs were detected using multiple-reaction monitoring mode of the transition of the ion [M–H]^−^ to the appropriate product ion. Masslynx 4.2 software was used to analyse the data and the standard isotope dilution method [43] was used to quantify GA levels.

### Transcript analysis

The peduncular node and 2 cm of attached peduncle from the primary and secondary tiller of glasshouse- and field-grown plants at heading stage were sampled in late morning and immediately frozen in liquid N_2_. Frozen tissues were ground in liquid N_2_ and stored at -80 °C. RNA was extracted from 80 mg subsamples of frozen tissue powder using a Monarch Total RNA Miniprep Kit (New England Biolabs) and assessed for concentration and quality using an Agilent Bioanalyser (Agilent, CA, USA). For qRT-PCR, 2.5 µg samples of RNA were reverse transcribed using Superscript IV (Thermo Fisher, MA, USA) with oligo-dT_17-20_ as primer; the resulting cDNA in a volume of 20 µL was diluted tenfold with RNAse-free water. Real-time qRT-PCR reactions used 4 µL diluted cDNA with 10 pmol of each primer in a total volume of 20 µL containing 10 µL JumpStart Taq ReadyMix (Sigma-Aldrich) and were run on a Quantstudio 1 (Thermo Fisher) with 40 data collection cycles at an annealing temperature of 60 °C. All primers were designed to amplify all three homoeologous copies of each target gene (Additional file 2, Table S2). Oligonucleotides were purchased from Sigma-Aldrich (MO, USA) or from MWG-Biotech (Ebersberg, Germany). Transcript levels were calculated using LinRegPCR v2021.1 [44] and relative values calculated against the geometric mean of the expression levels of three reference genes (*Ta2526*, *TaGAPD* and *TaSDH*) (Additional file 2, Table S2).

## Results

### Identification and characterisation of mutations in homoeologues of *GA20OX1* and *GA20OX2*

**Fig. 2:**
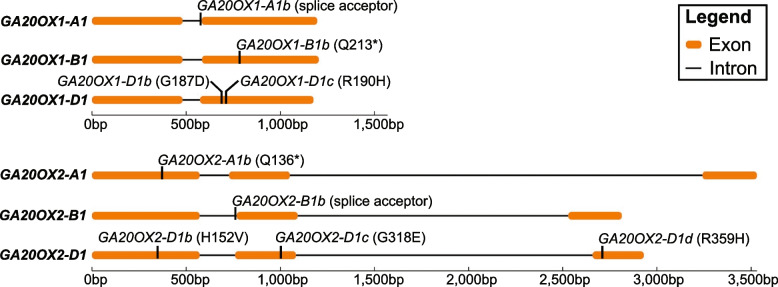
Locations of the induced mutations in *GA20OX1* and *GA20OX2* genes evaluated in this study. Sequences are drawn to scale with exons illustrated as orange bars and introns as black lines

We screened an ethyl methanesulphonate (EMS)-mutagenized wheat population using *Cel*I digestion and high-resolution melting analysis and identified lines carrying mutations introducing premature stop codons in *GA20OX1-B1* and *GA20OX2-A1*, mutations in the splice acceptor sites of *GA20OX1-A1* and *GA20OX2-B1*, and several mis-sense mutations in *GA20OX1-D1* and *GA20OX2-D1* predicted to have deleterious effects on the activity of the encoded enzyme (Fig. 2, Table 1 and Additional file 2, Table S3).

**Table 1:** Induced mutations in *GA20OX1* and *GA20OX2* evaluated in this study. Sorting Intolerant From Tolerant (SIFT) scores for amino acid substitutions in *GA20OX1-D1* and *GA20OX2-D1* are provided in brackets, where values less than 0.05 indicates the mutation is predicted to have a deleterious effect on protein function. ^*^indicates the introduction of a premature stop codon in the coding sequence

Gene	IWGSCv1.1 ID	Allele ID	Effect
*TaGA20OX1-A1*	*TraesCS4A02G319100*	*ga20ox1-A1b*	Splice acceptor
*TaGA20OX1-B1*	*TraesCS5B02G560300*	*ga20ox1-B1b*	Q213^*^
*TaGA20OX1-D1*	*TraesCS5D02G566200*	*ga20ox1-D1b*	G187D (0.02)
		*ga20ox1-D1c*	R190H (0.01)
*TaGA20OX2-A1*	*TraesCS3A02G406200*	*ga20ox2-A1b*	Q136^*^
*TaGA20OX2-B1*	*TraesCS3B02G439900*	*ga20ox2-B1b*	Splice acceptor
*TaGA20OX2-D1*	*TraesCS3D02G401400*	*ga20ox2-D1b*	H152V (0)
		*ga20ox2-D1c*	G318E (0)
		*ga20ox2-D1d*	R359H (0)

The *GA20OX1-B1b* and *GA20OX2-A1b* alleles carry point mutations resulting in the complete loss of enzyme activity as they both cause premature termination of translation at a position upstream of the Fe- and 2-oxoglutarate-binding residues in the GA20OX protein sequences that are essential for protein function (Additional file 1, Figure S2).

**Fig. 3:**
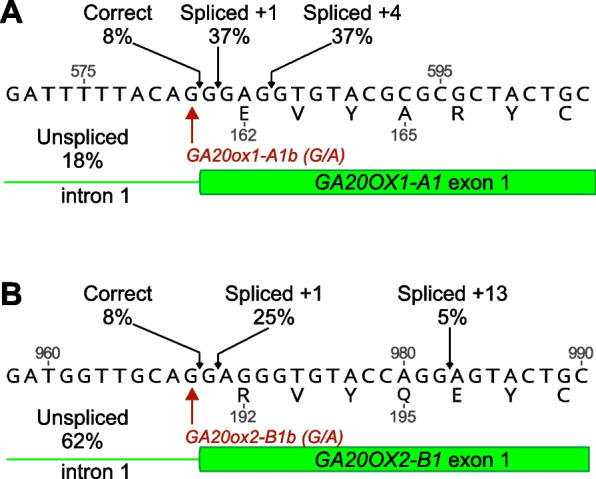
Proportion of correctly and incorrectly spliced transcripts in **A** *GA20OX1-A1b* and **B** *GA20OX2-B1b* alleles. Values are rounded to the nearest whole number. For *GA20OX1-A1b*, the lowest value was rounded up so that the total proportion equals 100%

Although mutations in conserved splice acceptor sites are assumed to result in aberrant splicing, latitude in the splicing mechanism or the presence of alternative, in-frame splice acceptor sites may result in the production of transcripts encoding a functional protein. To investigate the effects of the splice acceptor site mutations *GA20OX1-A1b* and *GA20OX2-B1b* (Fig. 2) on gene function we carried out deep sequencing of RT-PCR products spanning intron 1 of all homoeologues of the two genes, using cDNA generated from peduncular ligule tissue of single homozygous mutants and segregating wild-type lines (Fig. 3, Additional file 2, Table S4). In segregating wild-type plants, the majority of transcripts from all three homoeologues of *GA20OX1* and *GA20OX2* were correctly spliced, with at most 7% of transcripts being un-spliced. By contrast, in plants carrying the *GA20OX1-A1b* allele just 8% of transcripts from *GA20OX1-A1* were correctly spliced, whereas 74% were mis-spliced at positions + 1 or + 4 relative to the correct site, resulting in a translational frameshift (Fig. 3). The remainder of the transcripts (18%) were un-spliced (Fig. 3). Transcripts of all other *GA20OX1* or *GA20OX2* homoeologues in this mutant were correctly spliced as in the wild-type line (Additional file 2, Table S4). Similarly, in lines carrying the *GA20OX2-B1b* allele, 8% of transcripts from *GA20OX2-B1* were correctly spliced, while the majority (61%) of transcripts were un-spliced, with the remainder mis-spliced at positions + 1 and + 13 (Fig. 3, Additional file 2, Table S4).

For *GA20OX1-D1* and *GA20OX2-D1* we failed to find either non-sense or splice-site mutations (Additional file 2, Table S3). However, in each case we identified mis-sense mutations in conserved residues that Sorting Intolerant From Tolerant (SIFT) analysis suggested were highly deleterious (Fig. 2, Additional file 1, Figure S2, Table 1). To directly assess the effect of these mutations on enzyme activity we selected two mis-sense alleles of *GA20OX1-D1* (*GA20OX1-D1b* and *GA20OX1-D1c*) and three mis-sense alleles of *GA20OX2-D1* (*GA20OX2-D1b*, *GA20OX2-D2c* and *GA20OX2-D1d*) (Table 1). We replicated each mutation in a cDNA copy of the respective gene by synthesis and expressed the wild-type and mutant versions in *E. coli*. Cell lysates producing wild-type GA20OX1-D1 catalysed conversion of ^14^C-labelled GA_12_ to GA_9_, involving multiple oxidation steps and loss of C-20 (Additional file 1, Figure S3A). In contrast, expression products containing the mis-sense mutations *GA20OX1-D1b* and *GA20OX1-D1c* were almost completely inactive, producing only a small amount of GA_15_, with a single oxidation at C-20 (Additional file 1, Figure S3A). It was therefore concluded that both *GA20OX1-D1b* and *GA20OX1-D1c* encode null alleles of this gene. Due to similar results from heterologous expression and limited resources, only *ga20ox1-D1b* was taken forward into crosses with *GA20OX1-A1b* and *GA20OX1-B1b* to generate the *ga20ox1* mutant.

Despite numerous attempts to express *GA20OX2-D1* in *E. coli*, we could not demonstrate the full range of GA 20-oxidase activities for this protein: cell lysates from the wild-type GA20OX2-D1 converted GA_12_ only to GA_15_ (Additional file 1, Figure S3B). However, cell lysates from *E. coli* expressing cDNAs containing the mis-sense mutations *GA20OX2-D1b, GA20OX2-D1c* and *GA20OX2-D1d* had no detectable GA 20-oxidase activity (Additional file 1, Figure S3B), suggesting that these alleles should also be regarded as significantly impaired. All three alleles were taken forward for crossing with *GA20OX2-A1b* and *GA20OX2-B1b* alleles to develop three *ga20ox2* mutant lines with different *GA20OX2-D1* mutant alleles. Henceforth, we refer to these three mutant lines as *ga20ox2(b)*, *ga20ox2(c)* and *ga20ox2(d)*.

### Stacking mutations in *GA20OX1* and *GA20OX2* homoeologues

To generate *ga20ox1, ga20ox2(b), ga20ox2(c)* and *ga20ox2(d)* triple mutants, mutations in all three homoeologues were stacked and then backcrossed a minimum of three times to reduce the number of background EMS mutations (Additional file 1, Figure S1). Analysis of allele frequencies in the F_2_ generations of selfed triple heterozygotes (AaBbDd) in both *GA20OX* paralogues showed evidence of distorted segregation. All mutant alleles of *GA20OX1* and *GA20OX2* showed significantly reduced transmission, with the frequency of each mutant allele in the F_2_ generation being 8.1–15.7% lower than expected (Additional file 2, Table S5).

### Glasshouse phenotyping of *GA20OX1* and *GA20OX2* mutants

**Fig. 4:**
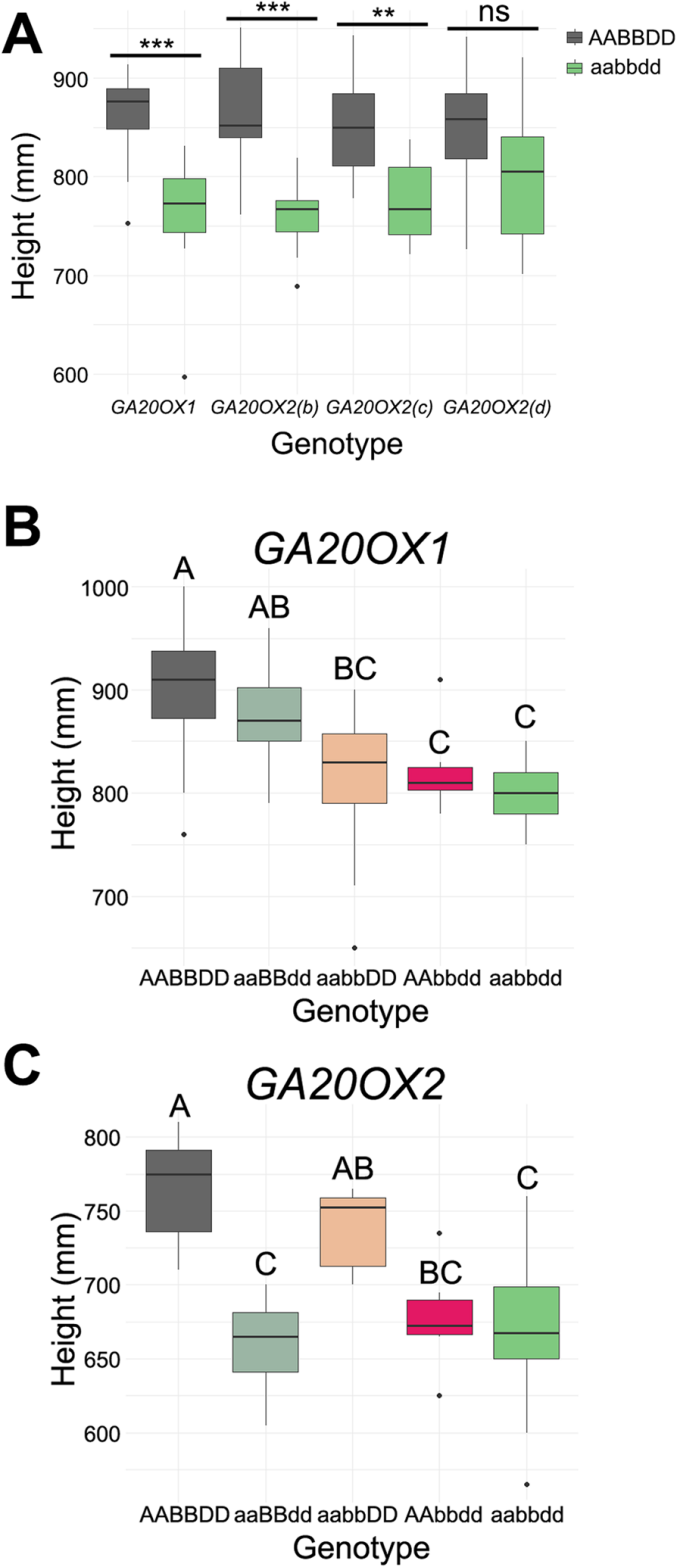
*GA20OX1* and *GA20OX2* mutations confer reduced height in glasshouse conditions. **A** Height of homozygous wild-type and triple mutant BC_5_F_3_ lines for each allelic combination. ** = *P* < 0.01, *** = *P* < 0.001 **B** Height of *ga20ox1* double and triple mutant combinations at BC_0_F_3_ generation. **C** Height of *ga20ox2(b)* double and triple mutant combinations at BC_0_F_2_ generation. In **B** and **C** genotypic classes that share a letter are not significantly different from one another (Tukey’s HSD, *P* > 0.05)

Initial phenotypic characterisation of the *ga20ox1* and *ga20ox2* triple mutants was performed in the glasshouse using homozygous lines at the BC_4-5_F_3_ or subsequent generation combined across backcrossing schemes (Additional file 1, Figure S1). Compared to their respective wild-type segregant, plant height was significantly reduced by 11.4% in the *ga20ox1* triple mutant (*P* < 0.001), by 12.0% in the *ga20ox2(b)* mutant (*P* < 0.001) and by 8.8% in the *ga20ox2(c)* mutant (*P* < 0.01) (Fig. 4A, Additional file 2, Table S6, Additional file 1, Figure S4A). Although the *ga20ox2(d)* mutant was 5.5% shorter than the wild-type segregant, this difference was not significant (*P* > 0.05) (Fig. 4A, Additional file 2, Table S6). Representative photos showing the height phenotypes of all lines are shown in Additional file 1, Figure S4A.

To determine the contribution of individual *GA20OX1* and *GA20OX2* homoeologues, we measured plant height in lines carrying different allelic combinations in segregating BC_0_F_2_ and BC_0_F_3_ populations in the glasshouse (Additional file 2, Table S7). For *GA20OX1*, all three double mutant combinations exhibited intermediate height between the wild-type and *ga20ox1* triple mutant, although only the aabbDD and AAbbdd double mutant lines were significantly shorter than the wild-type (*P* < 0.05) (Fig. 4B). The aaBBdd double mutant was significantly taller than the triple mutant (*P* < 0.05), suggesting that *GA20OX1-B1* is the most active *GA20OX1* homoeologue in developing wheat stems (Fig. 4B). However, in a public RNA-seq dataset from the variety ‘Azhurnaya’, *GA20OX1-B1* transcript levels are lower than *GA20OX1-A1* in peduncle and internode tissues at later stages of stem development (Additional file 1, Figure S5). In a population segregating for the *ga20ox2(b)* mutant alleles, the aaBBdd and AAbbdd double mutants were both significantly shorter than the wild-type (*P* < 0.05), but were not significantly different in height from the triple mutant (Fig. 4C). The aabbDD double mutant was not significantly different from the wild-type (*P* > 0.05) suggesting that *GA20OX2-D1* is the most active *GA20OX2* homoeologue in these tissues (Fig. 4C). This is consistent with expression data from the variety ‘Azhurnaya’ showing that *GA20OX2-D1* is the most highly expressed *GA20OX2* homoeologue in peduncle tissues at the grain milk stage, suggesting that differences in expression may account for differences in activity (Additional file 1, Figure S5).

There was evidence of delayed flowering in some lines: the *ga20ox1* and *ga20ox2(b)* triple mutants both headed approximately 3 days later than their corresponding wild-type segregants (*P* < 0.01) (Additional file 2, Table S6). However, heading date was not significantly different between *ga20ox2(c)* and *ga20ox2(d)* triple mutants and their respective wild-type segregants (*P* > 0.05) (Additional file 2, Table S6).

Taken together, these results suggest that both *GA20OX1* and *GA20OX2* contribute to wheat stem elongation in glasshouse conditions and that for both genes, all three homoeologues contribute additively to GA biosynthesis in stem tissues.

### Field phenotyping of *GA20OX1 *and *GA20OX2* mutants

**Fig. 5:**
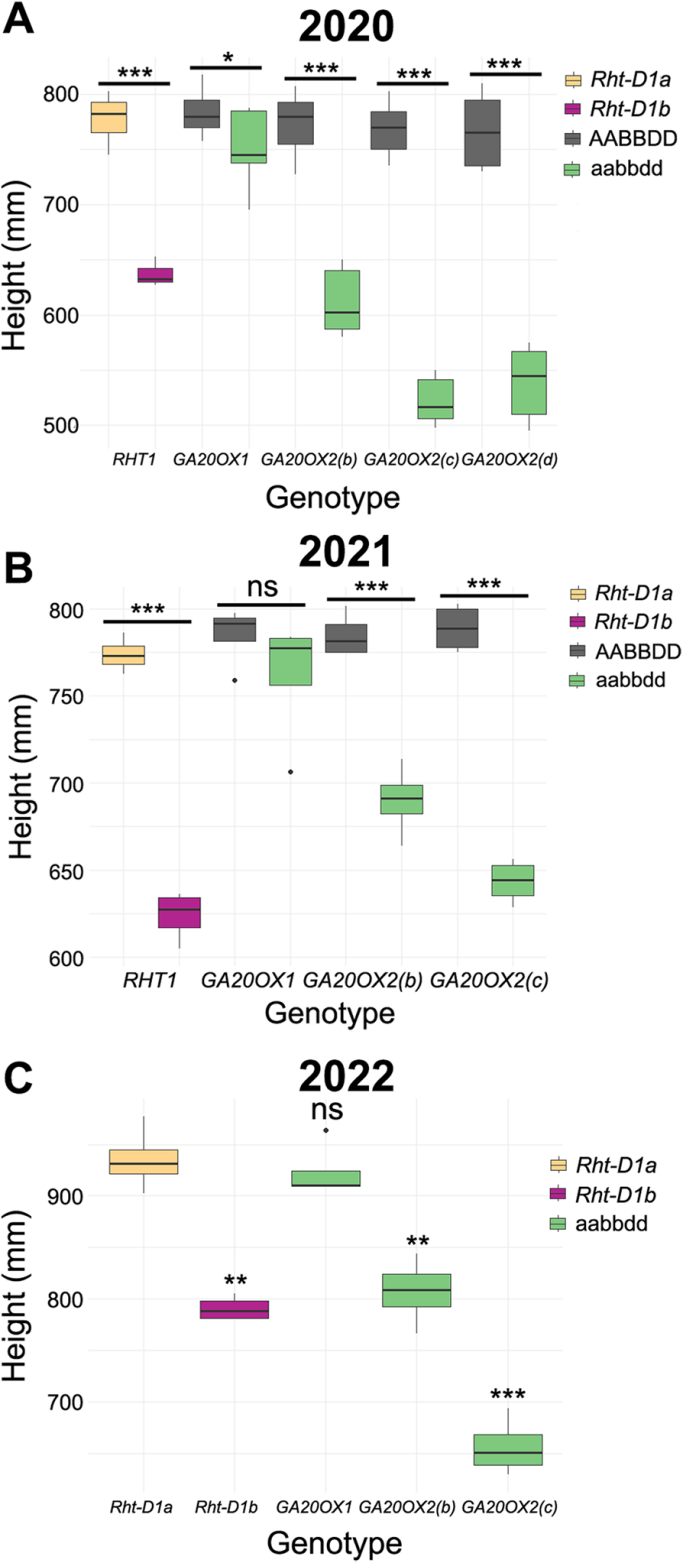
*GA20OX2* mutations confer reduced height in field conditions but *GA20OX1* mutations have only a minor effect on height. **A** Height of homozygous wild-type and triple mutant lines grown in small plots sown in spring 2020. **B** Height of homozygous wild-type and triple mutant lines grown in large plots sown in spring 2021. **C** Height of wild-type ‘Cadenza’ and homozygous triple mutant lines grown in large plots sown in autumn 2021 and evaluated in 2022. * = *P* < 0.05, ** = *P* < 0.01, *** = *P* < 0.001 based on two-tailed Student’s t-test

Initial field phenotyping was carried out in 1 m^2^ replicated plots sown in spring 2020, precluding accurate yield measurements. Within each genotype, plants from different backcrossing streams (three streams for *ga20ox1* and *ga20ox2(b)*, two streams for *ga20ox2(c)* and *ga20ox2(d)*) all showed similar height phenotypes (Additional file 2, Table S8), so we analysed the combined data across streams. The *ga20ox1* homozygous mutant conferred a 4.1% reduction in height and was significantly shorter than the wild-type segregating line (*P* < 0.05, Fig. 5A) but the difference was much smaller than that observed in glasshouse conditions (Fig. 4A). All three *ga20ox2* homozygous mutants conferred significant reductions in plant height of between 20.9% and 32.1% compared to their wild-type segregant lines (*P* < 0.001) (Fig. 5A, Additional file 2, Table S8). The reduction in height conferred by *ga20ox2* mutations was slightly greater than that of *Rht-D1b*, which was 17.9% shorter than the wild-type *Rht-D1a* allele in the ‘Cadenza’ background (*P* < 0.001) (Fig. 5A). Representative photos of all lines grown in field conditions are shown in Additional file 1, Figure S4B.

The seeds harvested from these plots were pooled by genotype and used to sow large-scale field experiments in spring 2021. The *ga20ox2(d)* mutant was not included in this experiment. The *ga20ox1* triple homozygous mutant was just 3.0% shorter than its wild-type segregant and the difference was not significant (*P* > 0.05) (Table 2, Fig. 5B). The *ga20ox2(b)* and *ga20ox2(c)* stacked mutations conferred significant reductions in height of 12.1% (*P* < 0.001) and 18.4% (*P* < 0.001) respectively, comparable to the 19.4% reduction conferred by the *Rht-D1b* allele (*P* < 0.001) (Table 2, Fig. 5B). Measurements of individual internodes showed that in both *ga20ox2(b)* and *ga20ox2(c)* mutants the three uppermost internodes, including the peduncle, were all significantly shorter than in their wild-type segregants and were affected approximately equally (Additional file 2, Table S9). By contrast, no stem internode was significantly different in length between the *ga20ox1* mutant and its segregating wild-type line (Additional file 2, Table S9).

**Table 2:**
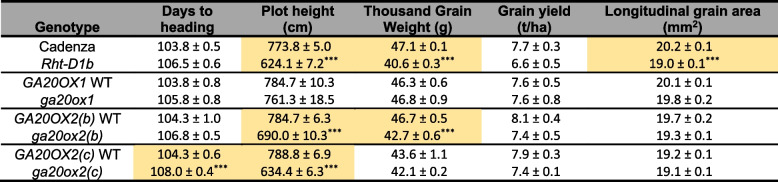
Phenotypic data of *Rht-D1b*, *ga20ox1 *and *ga20ox2 *mutations in the field in 2021. Grain yield measurements adjusted to 85% dry matter. Significant differences are highlighted in orange. *** = *P *< 0.001 contrasting the wild-type and mutant segregant lines from each genotype based on a two-tailed Student’s t-test

Heading date was delayed by between 2 and 4 days in all mutant lines compared to their respective wild-type segregants, although the difference was only significant for the *ga20ox2(c)* mutant (*P* < 0.001) (Table 2).

Grain yield from the *ga20ox2(b)* and *ga20ox2(c)* field plots were 9.0% and 6.4% lower than their respective wild-type plots, although these differences were not significant (Table 2). Yield was also reduced by 13% in *Rht-D1b* plots compared to ‘Cadenza’ wild-type plots, and marginally higher in the *ga20ox1* mutant, but neither of these differences were significant (Table 2). The *ga20ox2(b)*, *ga20ox2(c)* and *Rht-D1b* plots that had slight reductions in yield also exhibited reduced thousand grain weight (TGW), although the difference was significant only in *ga20ox2(b)* (*P* < 0.001) (Table 2). Longitudinal grain area was lower in the mutant alleles of all four genotypes but was only significantly different in *Rht-D1b* (*P* < 0.001) (Table 2).

To confirm these results, the field assessment was repeated as autumn-sown large-scale plots, but with the segregating wild-type lines omitted. Total height of the *ga20ox1* mutant was just 1.2% shorter than ‘Cadenza’ and was not significantly different (*P* > 0.05) (Table 3). Both *ga20ox2(b)* (13.7%, *P* < 0.01) and *ga20ox2(c)* (29.9%, *P* < 0.001) mutants were significantly shorter than ‘Cadenza’, comparable to the *Rht-D1b* mutant (15.5% shorter, *P* < 0.01) (Table 3). Consistent with the previous field evaluations, the dwarfing effect of *ga20ox2* and *Rht-D1b* mutations affected the lengths of the peduncle and upper three internodes approximately equally (Additional file 2, Table S10). Yields of *ga20ox1*, *ga20ox2(b)* and *Rht-D1b* plots were slightly higher than ‘Cadenza’, while the yield of *ga20ox2(c)* plots was slightly lower, but none of these differences were significant (*P* > 0.05; Table 3). TGW of *ga20ox1* and *Rht-D1b* were also slightly greater than ‘Cadenza’, but not significantly so, whereas TGWs of *ga20ox2(b)* and *ga20ox2(c)* were significantly reduced by 8.4% and 12.4%, respectively (Table 3). Reflecting this, grain area was also reduced by 4.4% in *ga20ox2(b)* mutants and by 5.5% in *ga20ox2(c)* mutants, but these differences were not significant (Table 3). Neither spikelet number, nor grain number per spike was significantly affected in *ga20ox1* or any *ga20ox2* mutant, suggesting these mutations have a minimal effect on inflorescence development. As in the spring-sown experiment, heading date was delayed by 2–3 days in *ga20ox1, ga20ox2(b)* and *Rht-D1b* mutants and by 7.5 days in the *ga20ox2(c)* mutant (Table 3).

**Table 3:**
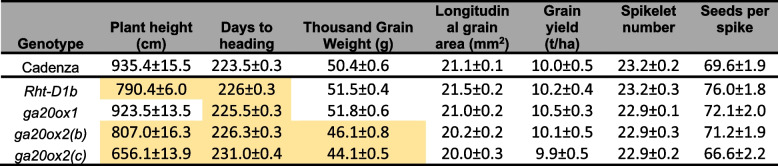
Field data from field experiment sown in autumn 2022. Results are means from 4 plots of each genotype +/- standard error of the mean. Values highlighted in orange indicate a significant difference from ‘Cadenza’ (*P *< 0.001) based on a two-tailed Student’s t-test

### Gibberellin and transcript quantification in *GA20OX1 *and *GA20OX2* mutants

Endogenous 13-OH and 13-H GAs were quantified in peduncles and peduncular nodes of the primary and secondary tillers of *ga20ox1, ga20ox2(b)* and *ga20ox2(c)* mutants and their respective wild-type lines grown in glasshouse and field conditions. In the glasshouse experiment, where both *ga20ox1* and *ga20ox2(b)* mutants exhibited reduced height (Fig. 4A), both mutants showed reductions in bioactive GA_1_ and GA_4_ levels compared to their wild-type segregants, although only the reduction in GA_4_ in the *ga20ox2(b)* mutant was significant (*P* < 0.05, Table 4). Both lines had significantly lower levels of the 2β-hydroxylated C_19_-GA GA_29_ (Table 4) and in the *ga20ox1* mutant, there was also a significant increase in the C_20_-GA substrate of GA 20-oxidase GA_19_ (*P* < 0.05, Table 4). In the 2021 field experiment, most GAs showed no significant changes in the *ga20ox1* mutant, consistent with the lack of a height phenotype in this environment (Fig. 5B). Levels of bioactive GA_1_ and GA_4_ were lower in both the *ga20ox2(b)* and *ga20ox2(c)* mutants compared to their wild-type segregants, although the difference was significant only for GA_1_ in the *ga20ox2(c)* mutant (*P* < 0.05, Table 4). Both mutants showed a significant increase in GA_53_, the C_20_ substrate of GA 20-oxidases (Table 4).

**Table 4:**
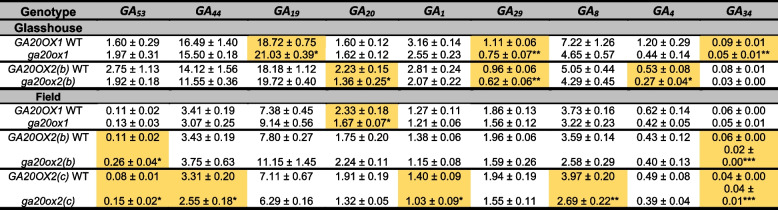
GA quantification in *GA20OX1 *and *GA20OX2 *wild-type and mutant genotypes in the glasshouse and field. Significant differences are highlighted in orange. * = *P *< 0.05, ** = *P *< 0.01, *** = *P *< 0.001 contrasting the wild-type and mutant segregant lines from each genotype

To investigate whether the phenotypes of *ga20ox1* and *ga20ox2* mutants might be affected by homeostatic regulation of GA biosynthesis, we quantified transcript levels of selected GA biosynthesis and signalling genes in elongating peduncles. In the glasshouse experiment, expression of *GA20OX1* was significantly reduced in the *ga20ox1* mutant compared to the ‘Cadenza’ control and, conversely, *GA20OX2* expression was lower in the *ga20ox2(b)* mutant, possibly reflecting the effects of the splice-site mutations in *ga20ox1-A1b* and *ga20ox2-B1b* and nonsense-mediated mRNA decay in *ga20ox1-B1b* and *ga20ox2-A1b* (Table 5). The *ga20ox2(b)* mutant also exhibited significantly higher transcript levels of *GA20OX4, GA3OX2* and *GID1* (*P* < 0.05, Table 5). These genes were also more highly expressed in the *ga20ox1* mutant, but the increases were smaller and not statistically significant (Table 5). In the 2021 field experiment, there were no significant changes in the target genes in the *ga20ox1* mutant, again correlating with its lack of a height phenotype in this environment (Table 4). In the *ga20ox2(b)* mutant, there was a small but significant increase in *GA20OX1* and *GA20OX4* expression and a decrease in *GA2OX3* expression.

**Table 5:**
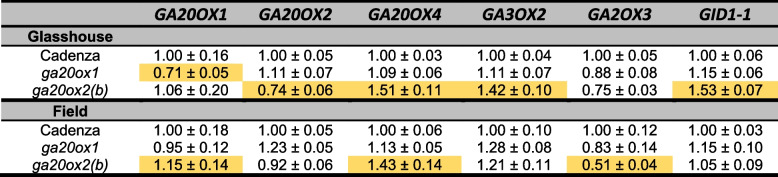
Transcript levels of selected GA biosynthetic and signalling genes in peduncle tissues of ‘Cadenza’, *ga20ox1 *and *ga20ox2(b) *mutants grown in glasshouse and field environments. Cells shaded in orange are significantly different from ‘Cadenza’ (*P *< 0.05). Relative transcript levels were calculated against the geometric mean of the expression levels of three reference genes and normalised to expression in ‘Cadenza’

## Discussion

### Reverse genetics in wheat

Overcoming functional redundancy in the polyploid wheat genome often requires the combination of null alleles in three homoeologous gene copies. Chemical mutagenesis is an inexpensive way to induce null alleles and mutagenised populations can be screened for alleles of interest using different approaches [45]. In the current study, we screened the wheat Cadenza-EMS population using *Cel*I-based TILLING and high-resolution melt analysis of genomic PCR products. These laborious methods limited the number of mutations detected, and we were able to identify null mutations introducing premature stop codons for only *GA20OX1-B1* and *GA20OX2-A1* (Table 1). Although the mutations in the intron 1 splice acceptor sites of *GA20OX1-A1* and *GA20OX2-B1* are located upstream of essential Fe- and 2-oxoglutarate-binding motifs in the GA20OX protein sequences (Additional file 1, Figure S2) both alleles exhibited a small proportion of correctly spliced transcripts (Fig. 3), meaning that neither can be regarded as a null allele.

For both *GA20OX1-D1* and *GA20OX2-D1* we identified only mis-sense mutations. The rice semi-dwarf variety ‘Calrose 67’ carries a point mutation introducing an L266F amino acid substitution in a highly conserved residue of the GA20OX2 protein that is just one residue downstream of the *GA20OX2-D1c* mutation selected in the current study (Additional file 1, Figure S2) [22]. However, while this mutation is sufficient to confer a semi-dwarf phenotype, the reduction in bioactive GA_1_ levels in ‘Calrose 67’ is proportionally less than in another semi-dwarf rice variety ‘Doongara’ which carries a 280 bp deletion in the *OsGA20OX2* gene body, suggesting the ‘Calrose 67’ allele retains some biochemical activity [22]. Heterologous expression of cDNAs containing the *GA20OX1-D1* and *GA20OX2-D1* mutations in the current study suggested that the expression products of all alleles were almost completely lacking in GA 20-oxidase enzyme activity (Additional file 1, Figure S3A), but it is also possible that low efficiency of expression or protein folding in this assay may have affected this result. Therefore, despite combining the most disruptive mutations identified, we cannot exclude the possibility that the homozygous wheat mutants we evaluated retain some GA biosynthetic activity.

Exons and promoters of the Cadenza-EMS population used in this work have now been sequenced, which uncovered more than 8 million EMS-type mutations [28, 46] that are searchable in silico on Ensembl plants (https://plants.ensembl.org/Triticum_aestivum). This database includes additional mutations introducing premature stop codons in *GA20OX1-A1* and *GA20OX1-B1*, and disrupting a splice-site in *GA20OX1-D1* that were not identified in our manual screen. However, no putative null alleles were identified in *GA20OX2-D1*, demonstrating that even within a large, sequenced population (1,521 sequenced lines) it is not always possible to identify null alleles for a target gene. Another drawback of chemical mutagenesis is that each line carries approximately 500,000 EMS-type mutations that can affect phenotypes in certain environments [28]. The effect of these mutations can be reduced by performing time-consuming backcrossing, but not eliminated.

Genome editing technologies, most notably CRISPR/Cas9, now allow the routine introduction of mutations in specific gene targets in plant genomes, overcoming some of these drawbacks. This approach has been used to improve lodging resistance in rice and Tef (*Eragrostis tef*) by inducing novel null alleles in *GA20OX2* orthologs [47, 48]. In wheat, CRISPR/Cas9 could be used to simultaneously induce null alleles in all three homoeologous copies of *GA20OX1* and *GA20OX2*. It is important to note that for most current applications of CRISPR/Cas9, a pre-requisite is that the target cultivar is amenable to transformation.

### *GA20OX1* plays a minor role in wheat stem elongation in field conditions

The *ga20ox1* triple mutant was, on average, 11.4% shorter than the wild-type segregant when evaluated in the glasshouse and had lower levels of bioactive GA_1_ and GA_4_ in peduncle tissues (Fig. 4A, Table 4). These results were not replicated in field conditions where height reductions in the *ga20ox1* mutant were much smaller (between 1.2 and 4.1%) and exhibited smaller reductions in bioactive GA_1_ levels (Fig. 5A-C, Table 4). The *GA20OX* genes are controlled by a wide range of environmental cues (reviewed in [49]) so it is possible that this discrepancy arises from exposure to different conditions in glasshouse and field environments. The effects of *GA20OX1* mutations on GA levels, and hence stature, may also be moderated by the feedback pathway that operates to regulate the later stages of the GA pathway via the GA response pathway [20], shown here by the upregulation of GA biosynthesis gene transcript levels in some mutants (Table 5). It would be interesting to combine *ga20ox1* and *ga20ox2* null mutations to determine whether *GA20OX1* plays a greater role in stem elongation in a *ga20ox2* background.

Our results across three years of evaluation consistently show that *GA20OX1* plays a minor role in wheat stem elongation in field conditions and is therefore unlikely to be a promising source of semi-dwarfing alleles for crop improvement. This contrasts with rice, where RNAi suppression of *OsGA20OX1* conferred a 13–18% height reduction [25]. Although these genes are orthologous, differences in the spatiotemporal expression profiles between species might account for this discrepancy [26]. This may also be reflected in the role of *OsGA20OX1* in rice panicle development as the underlying gene for the GRAIN NUMBER PER PANICLE locus [26, 27]. It is interesting to note that during population development, in the final self-fertilisation of the back-crossed, triple heterozygous mutants to generate homozygous lines we found significant segregation distortion of all the alleles employed (Additional file 2, Table S5). This implies that individual loss-of-function or reduced function alleles of *GA20OX1* have reduced fitness, potentially due to reduced GA levels during a key stage in reproductive development. However, we observed no significant difference in spikelet number or grain number in the *ga20ox1* mutant (Table 3) nor major differences in heading date (Table 2), suggesting *GA20OX1* plays a limited role in wheat inflorescence development, in contrast to the orthologous gene in rice.

### *GA20OX2* null mutations reduce height in wheat and may be useful semi-dwarfing alleles

In both glasshouse and field environments, mutations in *GA20OX2* consistently confer a reduction of height ranging from 12.1% in the *ga20ox2(b)* mutant to 32.1% in the *ga20ox2(c)* mutant. This effect on stature is similar to that of null or reduced-function alleles of *SD1*, the orthologous rice gene [21, 22], which in different cultivars confers a height reduction of 19–38% [50]. Similarly in barley, a number of alleles of the *denso/sdw1* gene, which encodes *HvGA20OX2* [51, 52], confer semi-dwarfism, minimising lodging while maintaining yield and having no deleterious effects on grain size [53, 54]. For example, the *sdw1.d* allele that contains a 7-bp deletion in the first exon of *HvGA20OX2*, confers a reduction in height of approximately 10% and is deployed in both malting and feed varieties [54]. These observations suggest that *GA20OX2* genes play a conserved role in regulating cell elongation in vegetative tissues across monocots.

Our results suggest that *GA20OX2* homoeologues contribute additively to GA biosynthesis in the developing stem (Fig. 4C) and that the selection of different combinations of null alleles could confer a range of height reduction that might be beneficial in some environments. However, in most cases it is likely that a line carrying loss-of-function mutations in all three homoeologues will be most useful for growers, which would require the selection and maintenance of three independent alleles during variety development. This contrasts with the gain-of-function *Rht-B1b* and *Rht-D1b* alleles which confer a comparable height reduction from a single allele.

Stacked mutations in *GA20OX2* thus confer a semi-dwarf phenotype that might be useful in wheat breeding as protection against lodging in the field. In the two years of large-scale field experiments grain yield was maintained for both the *ga20ox2(b)* and *ga20ox2(c)* mutant lines compared with their wild-type segregants, despite a decrease in TGW (Tables 2 and 3). It should be noted that the background germplasm ‘Cadenza’ is a “short tall” spring type containing no *Rht1* dwarfing alleles but possessing a number of height reducing QTLs [55], and further height reduction may not necessarily increase grain yields. This was reflected in our results showing that *Rht-D1b* did not improve yields in this background (Tables 2 and 3). It therefore will be important to evaluate these novel *GA20OX2* alleles in a range of more vigorous winter background genotypes, including those susceptible to lodging, to help determine their potential value in wheat breeding.

## Conclusions

Our study demonstrates that induced mutagenesis can expand genetic variation in polyploid crops to uncover novel alleles that may be beneficial for breeding. We found that *GA20OX1* plays a minor role in stem elongation in field conditions and that mutations in *GA20OX2* could have utility in wheat breeding programmes as alternative semi-dwarfing alleles.

### Supplementary Information

Supplementary Material 1.

Supplementary Material 2.

## Data Availability

All data generated in this study are included in this published article and its supplementary information files. Biological materials are available upon request from the corresponding author. Details of all the genes described in this study are provided in Additional file 2, Table S1.

## Abbreviations

EMS: Ethyl MethaneSulphonate
GA: Gibberellin
HRM: High Resolution Melt
KASP: Kompetitive Allele-Specific PCR
SIFT: Sorting Intolerant From Tolerant
TGW: Thousand Grain Weight
