# Supplementary material for: Stacked mutations in wheat homologues of rice SEMI-DWARF1 confer a novel semi-dwarf phenotype
Source: BMC Plant Biol. 2024 May 9;24:384. doi: 10.1186/s12870-024-05098-1 (PMC11080193; doi:10.1186/s12870-024-05098-1)

Stacked mutations in wheat homologues of rice *SEMI-DWARF1* confer a novel semi-dwarf phenotype

Barbora Ndreca[1], Alison Huttly[1], Sajida Bibi[1,2], Carlos Bayon[1], George Lund[1], Joshua Ham[1], Rocío Alarcón-Reverte[1], John Addy[1], Danuše Tarkowská[3], Stephen Pearce[1,*], Peter Hedden[1,3], Stephen G. Thomas[1] & Andrew L. Phillips[1]

1. Rothamsted Research, Harpenden, Hertfordshire, AL5 2JQ, UK.
2. Nuclear Institute for Agriculture and Biology, Faisalabad, Punjab, Pakistan.
3. Laboratory of Growth Regulators, Institute of Experimental Botany, Czech Academy of Sciences and Palacky University, Šlechtitelů 27, CZ-78371 Olomouc, Czech Republic.

* Corresponding author.

Additional file 1 (PDF) Figures S1 – S5.

Figure S1: Crossing strategy to develop *ga20ox1*, *ga20ox2(b)*, *ga20ox2(c)* and *ga20ox2(d)* triple mutants.

Figure S2: Alignment of *GA20OX1* and *GA20OX2* amino acid sequences from wheat, rice, and *Arabidopsis*.

Figure S3: Activity of *GA20OX1-D1* and *GA20OX2-D1* alleles in heterologous expression.

Figure S4: Representative pictures of plants carrying wild-type (W) and three homoeologous mutations (M) in *ga20ox1* and *ga20ox2* target genes.

Figure S5: Transcript levels of *GA20OX1* and *GA20OX2* in peduncle and internode 2 tissues at three stages of development.

Figure S1: Crossing strategy to develop *ga20ox1*, *ga20ox2*(*b*), *ga20ox2*(*c*) and *ga20ox2*(*d*) triple mutants.

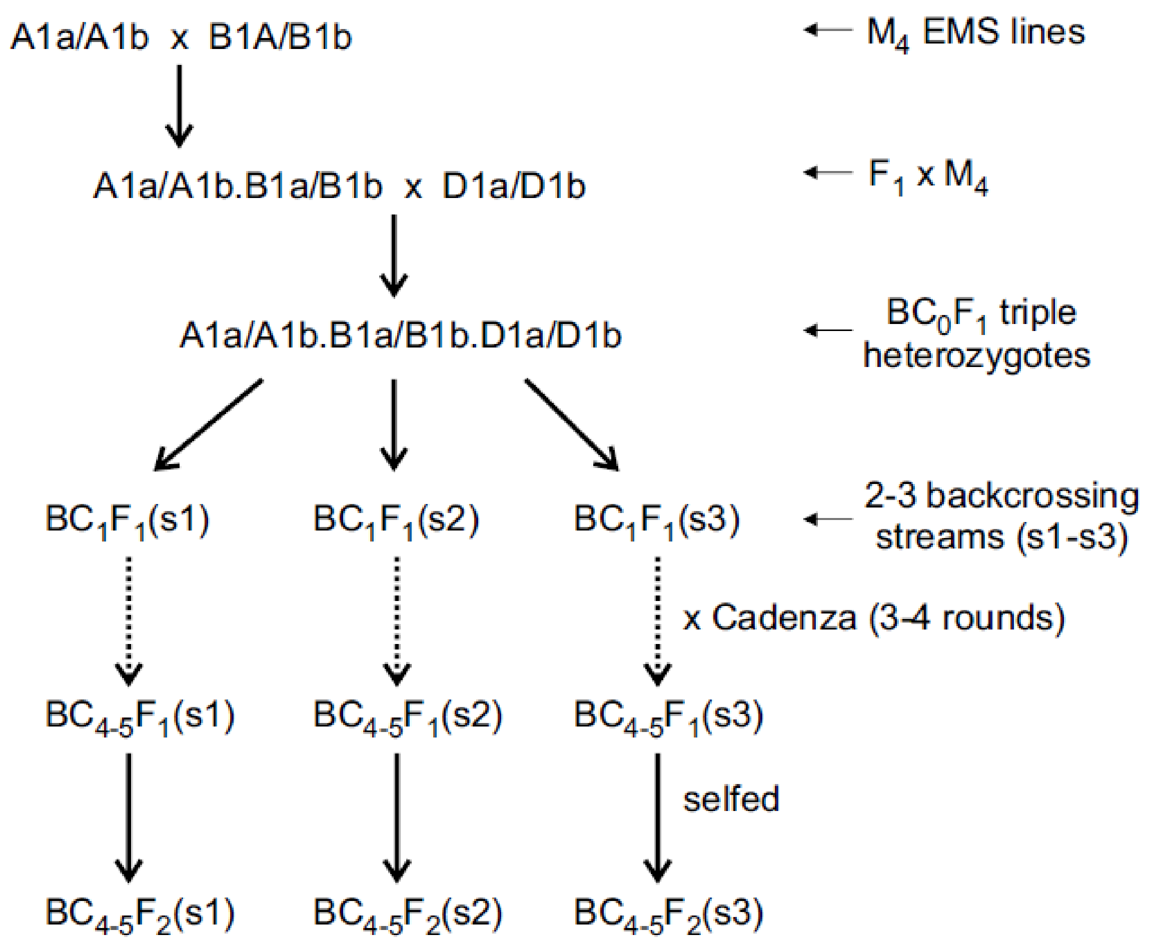

Figure S2: Alignment of GA20OX1 and GA20OX2 amino acid sequences from wheat, rice and *Arabidopsis*. The position of mutations in wheat genes evaluated in this study are labelled. The red caret indicates the position of the L266F point mutation in *OsGA20OX2* in the rice variety 'Calrose 67'. Red and green asterixis indicate conserved Fe- and 2-ODD binding residues, respectively.

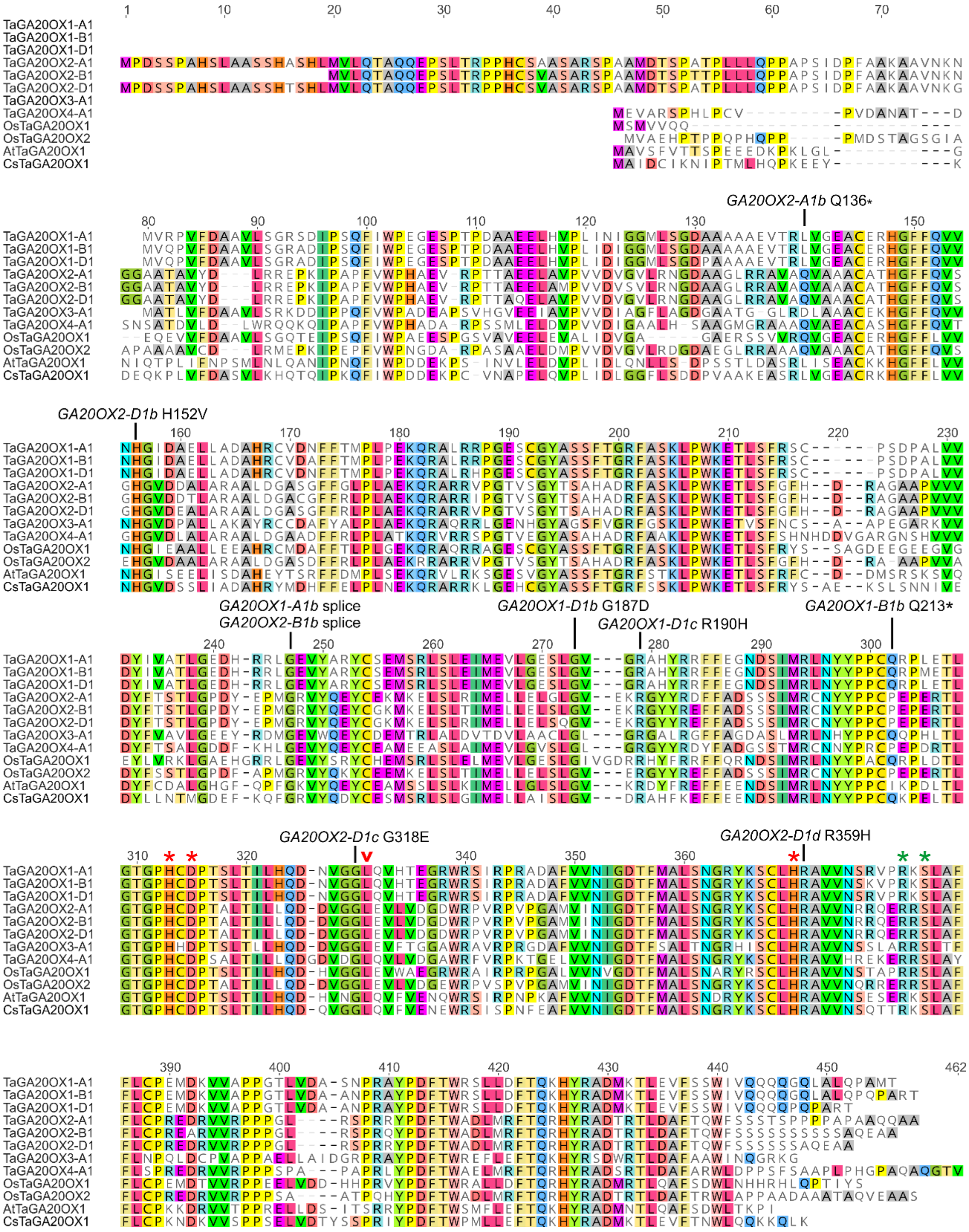

Figure S3: Activity of (A) GA20OX1-D1 and (B) GA20OX2-D1 alleles in heterologous expression.

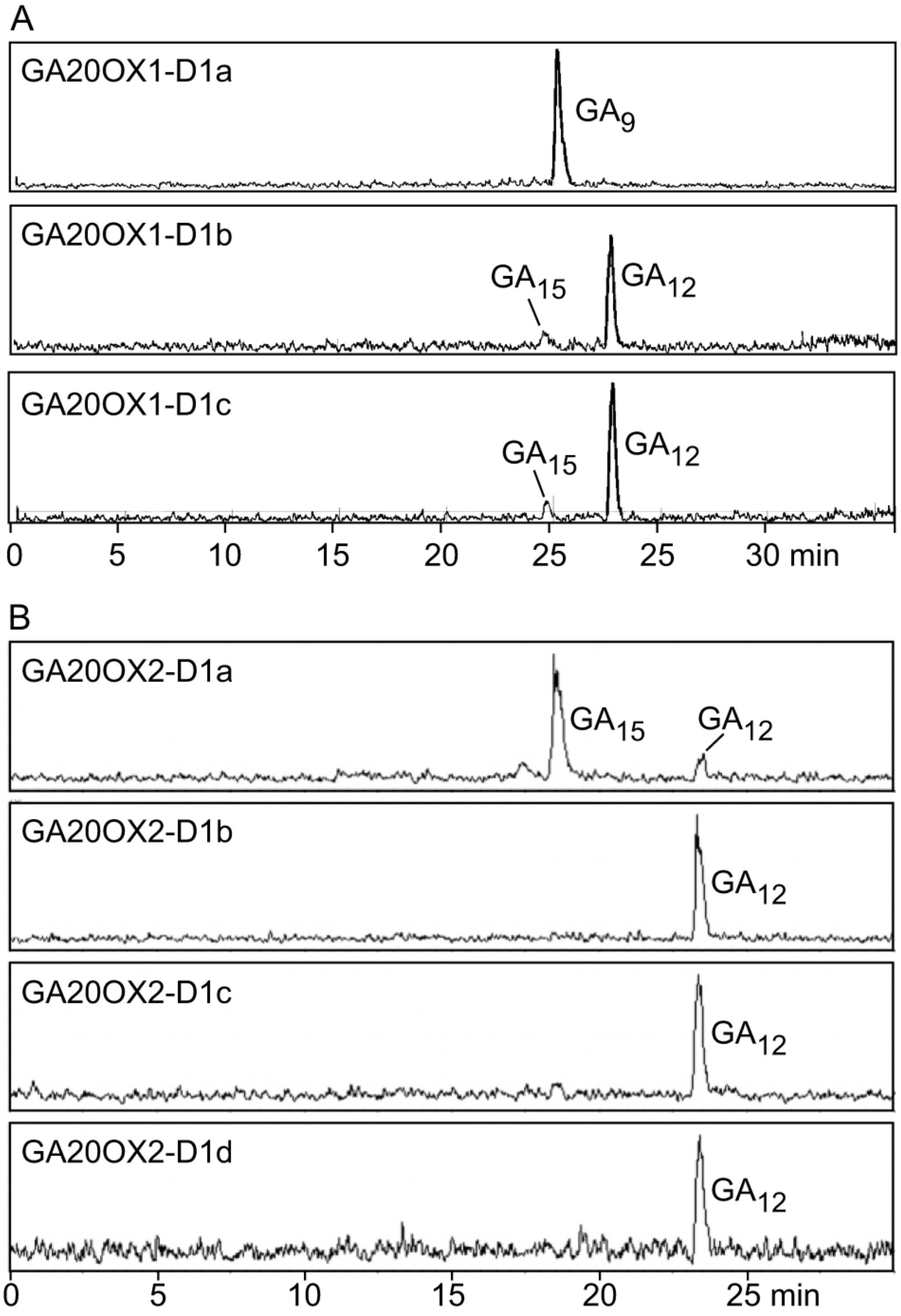

Figure S4: Representative pictures of plants carrying wild-type (W) and three homoeologous mutations (M) in *ga20ox1* and *ga20ox2* target genes. Plants were grown in (A) greenhouse and (B) field conditions. Wild-type 'Cadenza' and *Rht-D1b* are included as a comparison.

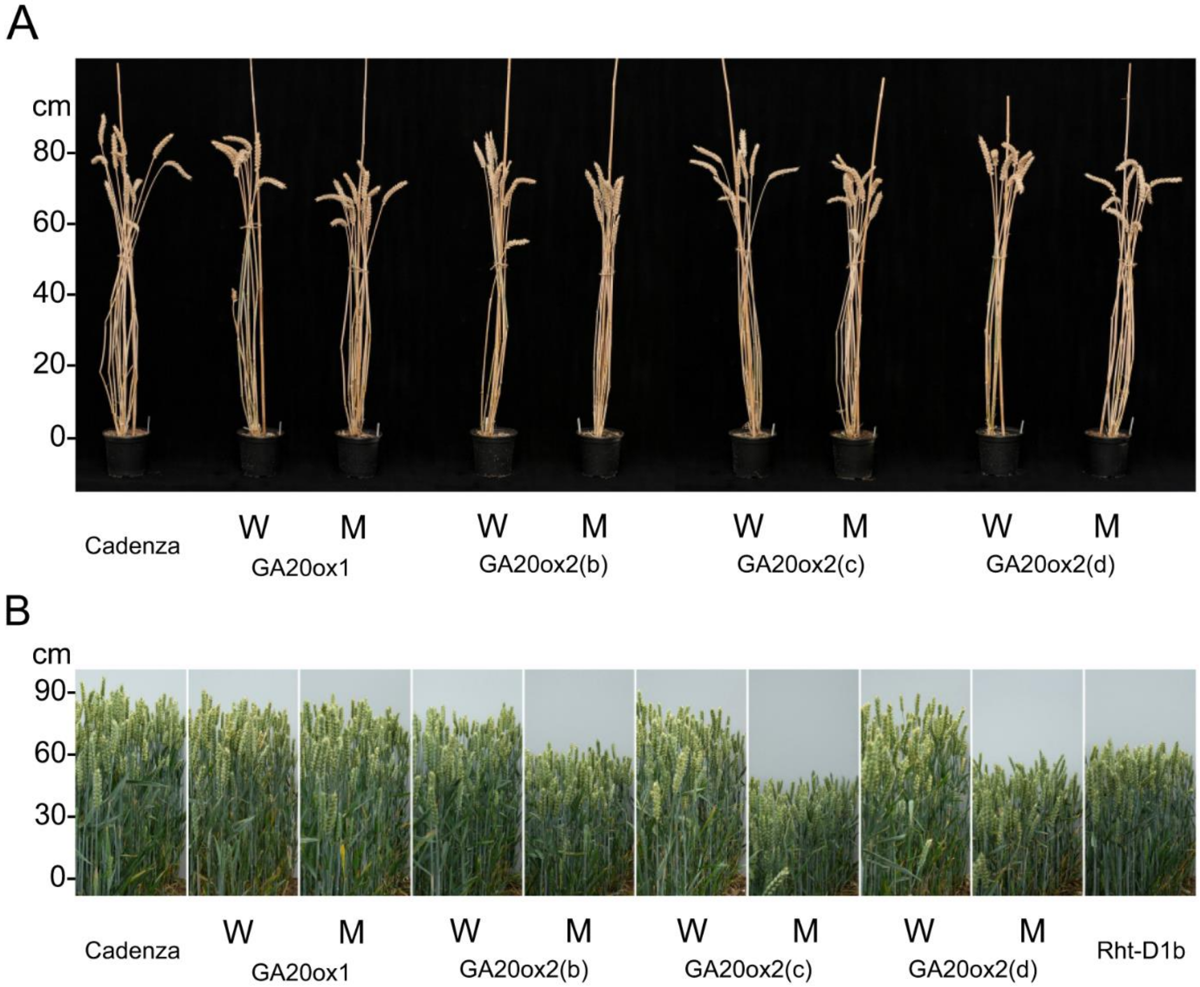

Figure S5: Transcript levels of *GA20OX1* and *GA20OX2* in peduncle and internode 2 tissues at three stages of development in the variety 'Azhurnaya'. Expression data was extracted from the dataset described by Ramirez-Gonzalez *et al*. 2018) and presented as the mean TPM ± standard error (n = 3).

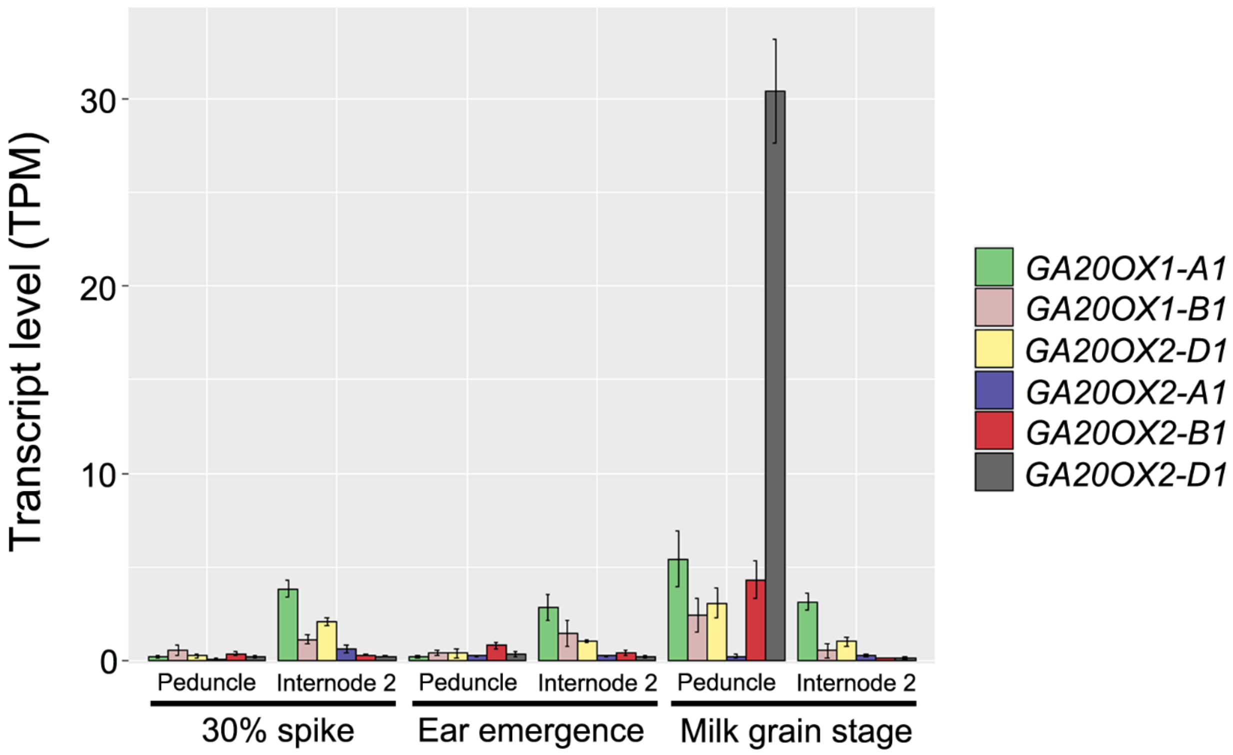

Supplement: Supplementary file 1 — Supplementary Material 1. [file 12870_2024_5098_MOESM1_ESM.pdf]
